# Mendelian Randomization Revealed Potential of mTOR Inhibitors for Treatment of Osteoporosis: Evidence From GWAS and Transcriptome Data

**DOI:** 10.1155/ije/5562148

**Published:** 2026-05-23

**Authors:** ZhaoLiang Zhang, LiMing Zhou, LieHui Yao, Tao Ma, YunFei Xu

**Affiliations:** ^1^ 108th Division, Orthopaedic Centre, The Affiliated YiXing Hospital of Jiangsu University, YiXing, 214200, JiangSu, China, ujs.edu.cn

**Keywords:** drug targets, GWAS, Mendelian randomization, mTOR, osteoporosis, PheWAS, transcriptome

## Abstract

**Aims:**

Numerous preclinical studies suggested that targeted inhibition of mammalian target of rapamycin (mTOR) may be beneficial for the treatment of osteoporosis. However, the relevance of these findings to human populations remains unclear. We hypothesized that lifelong genetically higher mTOR expression leads to reduced bone mineral density (BMD), and we use Mendelian randomization to estimate its causal effect.

**Background:**

We aimed to utilize existing genome‐wide association studies (GWAS) and expression quantitative trait locus (eQTL) data to comprehensively evaluate whether genetically proxied mTOR expression is associated with BMD using the Mendelian randomization method.

**Methods:**

Independent cis‐eQTLs for the gene‐encoding mTOR were selected as instrumental variables (IVs). Two large GWAS meta‐analysis cohorts were used for BMD outcomes. Summary data–based Mendelian randomization (SMR), inverse variance–weighted Mendelian randomization (IVW‐MR), and multivariable Mendelian randomization (MVMR) were applied to estimate the association between genetically proxied mTOR expression and BMD. Phenome‐wide association study (PheWAS) analysis was conducted to explore potential horizontal pleiotropy and genetically associated phenotypes related to mTOR. Bayesian colocalization was performed to assess whether mTOR expression and BMD shared a common causal single‐nucleotide variant (SNV). Blood transcriptome data were analyzed to further evaluate expression patterns of mTOR in relation to BMD status. In addition, molecular docking was conducted to explore potential interactions between mTOR and representative fatty acids. Analyses followed STROBE‐MR guidance.

**Results:**

SMR analysis indicated that increased genetically proxied mTOR expression in blood was associated with reduced BMD (OR = 0.8130–0.9340), except in individuals younger than 30 years. Colocalization analysis suggested that mTOR expression and BMD may share a common genetic locus (posterior probability for H4 = 77.7%). PheWAS identified an association between mTOR and basal metabolic rate (Beta = −16.769, FDR = 2.21E − 25), indicating potential metabolic implications. Further analyses evaluated associations between mTOR‐related genetic variation, circulating fatty acids, hormone levels, and BMD. MVMR results suggested that in individuals aged 30–45 years, the association between genetically proxied mTOR expression and BMD may be influenced by blood fatty acid levels (OR = 0.8823–0.9020). In older age groups (45–60 years and ≥ 60 years), genetically predicted insulin levels were associated with attenuation of these metabolic effects. Blood transcriptome analysis showed no significant difference in mTOR expression between high‐ and low‐BMD groups, indicating that peripheral transcript levels were not detectably associated with BMD status. Arachidonic acid demonstrated the highest predicted binding affinity to mTOR (−5.9 kcal/mol), followed by palmitic acid (−5.0 kcal/mol), oleic acid (−4.9 kcal/mol), linoleic acid (−4.8 kcal/mol), and stearic acid (−4.4 kcal/mol). Given the modest binding energies and lack of experimental validation, these findings should be interpreted cautiously and are intended to provide preliminary mechanistic hypotheses rather than confirm functional interactions.

**Conclusions:**

Genetic evidence indicates that mTOR‐related pathways may contribute to variation in BMD, with age‐dependent metabolic associations. The molecular docking results provide exploratory structural hypotheses that may inform future mechanistic studies but do not constitute functional or translational validation. Although these findings do not establish the clinical efficacy of pharmacological mTOR inhibition, they offer human genetic evidence supporting further experimental and pharmacological investigation of mTOR as a potential therapeutic target in osteoporosis.

## 1. Background

Osteoporosis is a metabolic bone disorder characterized by reduced bone mineral density (BMD) and deterioration of bone microarchitecture, representing a major global public health concern [1]. Worldwide, osteoporosis is associated with disability, impaired quality of life, chronic pain, and substantial healthcare costs, particularly among older adults [2, 3]. With progressive population aging, the incidence of osteoporotic fractures has increased markedly in recent decades [2, 3]. Although multiple pharmacological therapies are available, long‐term effectiveness remains suboptimal in a considerable proportion of patients [4].

The mammalian target of rapamycin (mTOR) is a serine/threonine kinase that regulates cellular growth, proliferation, and senescence through multiple signaling pathways and was initially investigated in the context of tumor metabolism [5, 6]. Emerging evidence indicates that the mTOR signaling pathway is also implicated in various nonmalignant conditions, including arthritis, diabetes, and osteoporosis [6]. Experimental and clinical studies have suggested that pharmacological mTOR inhibitors, such as rapamycin, may influence bone homeostasis [7]. For example, Luo et al. [8] reported that intraperitoneal administration of rapamycin attenuated age‐related bone loss in rats. Campistol et al. [9] observed higher osteogenic markers in renal transplant recipients treated with sirolimus compared with cyclosporine. However, these studies are subject to limitations, including potential reverse causation, limited sample sizes, and insufficient long‐term follow‐up. Moreover, given the highly conserved and pleiotropic nature of the mTOR pathway, systemic inhibition may be associated with unintended effects, which require further evaluation in well‐designed clinical studies [6].

Genetic approaches provide an alternative strategy to investigate the relationship between drug targets and disease phenotypes [10, 11]. Large‐scale genome‐wide association studies (GWAS) and expression quantitative trait locus (eQTL) datasets enable the assessment of associations between genetically predicted gene expression and complex traits. Mendelian randomization (MR) leverages genetic variants, such as cis‐eQTLs, as instrumental variables (IVs) to estimate the association between lifelong genetically proxied gene expression and disease outcomes. Importantly, genetically proxied mTOR expression reflects inherited variation in gene regulation and does not directly model pharmacological mTOR inhibition, which is dose‐dependent, tissue‐specific, and temporally limited. Nevertheless, genetic evidence can help prioritize potential therapeutic targets and reduce the confounding inherent in observational studies [12]. Such approaches may also inform subsequent experimental and clinical research while minimizing unnecessary exposure during early‐stage drug development [1].

In this study, we aimed to systematically evaluate the association between genetically proxied mTOR expression and BMD. Bayesian colocalization analysis was performed to assess whether mTOR expression and BMD share common genetic variants. As a complementary analysis, a phenome‐wide association study (PheWAS) was conducted to explore broader phenotype associations potentially related to mTOR. We additionally examined peripheral blood transcript levels of mTOR in relation to BMD status. Finally, molecular docking analysis was performed as an exploratory, hypothesis‐generating approach to assess potential structural interactions between mTOR and selected fatty acids. By integrating multiple genetic and computational approaches, this study provides human genetic evidence relevant to the biological role of mTOR in bone metabolism and may inform future mechanistic and pharmacological investigations.

## 2. Methods

### 2.1. Acquisition of Genetic IVs

This study employed summary‐level data to conduct MR analysis. Genetic instruments for mTOR expression in blood were obtained from the eQTLGen consortium and the GTEx project, while BMD outcomes were derived from large‐scale BMD GWAS data in the UK Biobank cohort and a whole‐body BMD meta‐analysis by Medina‐Gomez et al. [13–20]. Transcriptome sequencing studies were used to measure mTOR expression levels in blood [21]. PheWAS analyses were conducted using the genetic association results reported by Canela‐Xandri et al., based on 118,660 European‐ancestry participants from the UK Biobank cohort [22]. Genetic data for mTOR expression in blood were obtained from eQTLGen (*N* ≈ 31,000) and GTEx V8 (*N* ≈ 670). Heel BMD was measured using quantitative ultrasound (QUS) in the UK Biobank, and whole‐body BMD was obtained from the meta‐analysis by Medina‐Gomez et al. (measured using DXA). PheWAS was conducted based on 118,660 European‐ancestry individuals from the UK Biobank. Detailed characteristics of all included datasets are provided in Supporting Table 1. All original studies had obtained appropriate ethical approval.

### 2.2. Summary Data–Based Mendelian Randomization (SMR) Analysis

We selected cis‐eQTLs for mTOR (minor allele frequency [MAF] > 0.01) from the eQTLGen Consortium (https://www.eqtlgen.org/) and GTEx Consortium V8 (https://gtexportal.org/) as genetic instruments for mTOR expression (Supporting Table 3). The SMR method was applied to evaluate the association between genetically proxied mTOR expression and complex traits using GWAS and eQTL summary statistics [23].

GWAS summary‐level data for heel BMD were obtained from individuals of European ancestry in the UK Biobank. Age‐stratified total body BMD meta‐analysis data reported by Soranzo et al. [19] were used for external validation. SMR analyses were conducted using SMR software Version 1.3.1 (https://yanglab.westlake.edu.cn/software/smr/%23Download). The estimated effect sizes represent the association between genetically predicted mTOR expression and BMD, rather than the pharmacological effects of mTOR inhibition.

### 2.3. Bayesian Colocalization of mTOR and BMD

Bayesian colocalization analysis was performed to evaluate whether two phenotypes are likely driven by the same causal variant within a genomic region, under five mutually exclusive hypotheses (H0–H4). This approach was used to assess the consistency of the SMR findings. A 1‐Mb region centered on the mTOR cis‐eQTLs was defined for the analysis. The “coloc.abf” function from the *R* package coloc was applied to estimate the posterior probability that mTOR expression and heel BMD share a common causal variant (H4). A posterior probability for H4 (PP.H4) greater than 0.75 was considered suggestive evidence of colocalization within the specified region [24].

### 2.4. PheWAS and Inverse Variance–Weighted Mendelian Randomization (IVW‐MR) Analysis

To further explore potential pleiotropic associations related to mTOR, we examined the association between the top cis‐eQTL for mTOR and 118 continuous as well as 660 binary traits using GeneATLAS (https://geneatlas.roslin.ed.ac.uk) [18]. Resulting *p* values were adjusted for false discovery rate (FDR), with thresholds set at FDR < 0.05 and |Beta| > 0.1 to reduce the likelihood of false‐positive findings.

IVW‐MR and multivariable Mendelian randomization (MVMR) were applied to evaluate the associations between genetically proxied mTOR expression, identified phenotypes, and BMD. For each exposure–outcome pair, MR analyses were conducted using the “TwoSampleMR” *R* package (Version 0.5.6) [25], with IVW as the primary analytical method. For phenotypes potentially associated with BMD, MVMR was performed to estimate their joint associations with BMD across different age groups.

### 2.5. Blood Transcriptome Analysis

Peripheral blood transcriptome sequencing datasets from individuals with low and high BMD (GSE56814 and GSE56815) were obtained from the Gene Expression Omnibus (GEO) database (https://www.ncbi.nlm.nih.gov/geo/) [26]. The *R* packages “GEOquery” [27] and “Bioconductor” [28] were used to retrieve expression matrices, gene annotation files, and corresponding clinical information. Probe identifiers were mapped to gene symbols according to the annotation files, and probes without annotated gene names were excluded. When multiple probes corresponded to the same gene, the probe with the maximum expression value was retained.

Batch effects between GSE56814 and GSE56815 were corrected using the “ComBat” function from the *R* package “sva,” while the “normalizeBetweenArrays” function from the “limma” package was applied for between‐sample normalization [29]. Boxplots and principal component analysis (PCA) plots indicated that most batch effects were effectively reduced (Supporting Figure 1). Differential expression analysis of mTOR between low‐ and high‐BMD groups was performed using the “limma” package.

### 2.6. Molecular Docking Analysis

Molecular docking simulations were performed to explore potential structural interactions between selected molecules and mTOR. The molecular structures of candidate compounds were retrieved from the PubChem database (https://pubchem.ncbi.nlm.nih.gov/) in SDF format. The three‐dimensional crystal structure of mTOR was obtained from the Protein Data Bank (https://www.rcsb.org/) in PDB format. Protein preprocessing, including the removal of water molecules and cocrystallized ligands, was conducted using PyMOL, and the prepared structure was saved in PDB format. Docking grid parameters were defined using the Getbox plugin. The processed protein and ligand structures were imported into AutoDock Tools 1.5.6 and converted into PDBQT format. Molecular docking was performed using AutoDock Vina 1.1.2, and docking poses were visualized using PyMOL 2.6.0. Binding energy (kcal/mol) was used as the primary metric to estimate the relative binding affinity between mTOR and the candidate molecules.

### 2.7. Statistical Method and Sensitivity Analysis

Continuous BMD values were log‐transformed to calculate SMR regression results, and odds ratios (ORs) were reported in exponentiated form, representing relative BMD changes per standard deviation change in mTOR expression. The exposure (mTOR expression) was analyzed per SD unit, and BMD outcomes were treated as continuous variables, with SMR regression coefficients exponentiated to report ORs reflecting relative BMD changes. Age‐stratified groups were defined as < 30, 30–45, 45–60, and > 60 years. The primary analysis used SMR software (v1.3.1) to estimate the association between mTOR expression and BMD, while IVW‐MR and MVMR analyses were conducted using the TwoSampleMR *R* package (v0.5.6). Age‐stratified IVW‐MR analyses were performed to estimate effect sizes in different age groups. MVMR simultaneously included genetic instruments for mTOR and metabolic markers to assess the independent effects of each exposure. For IVW‐MR, MR‐PRESSO was used to remove SNPs that might have horizontal pleiotropy by *R* package “MRPRESSO” [30]. Heterogeneity was tested by the Rucker *Q* statistic (MR‐Egger), the I2 index, and Cochran’s *Q* statistic (IVW) [25]. *F* values were calculated to evaluate the statistical efficacy of SNPs [31]. The HEIDI test was used to test whether the results of the SMR are influenced by linkage disequilibrium. The study was conducted in accordance with STROBE‐MR guidelines, with the detailed checklist provided in the Supporting list.

**FIGURE 1:**
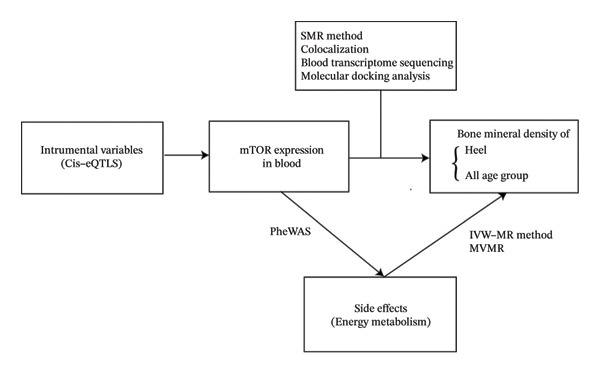
Flowchart.

The flowchart is shown in Figure 1.

## 3. Results

### 3.1. Genetically Proxied mTOR Expression in Blood Was Negatively Correlated With Heel BMD and Total Body BMD in 30–60 Years of Age

In the SMR analyses, rs2295079 was identified as the top SNP for mTOR expression in the GTEx cohort, whereas rs4845985 was the top SNP in the eQTLGen cohort (Supporting Table 3). SMR results (Figure 2(a); Supporting Table 2) demonstrated that genetically predicted higher blood mTOR expression (per SD increase) was significantly associated with lower heel BMD (OR = 0.9284 in GTEx; 0.9340 in eQTLGen; *p* = 9.22E − 07 and 1.81E − 08, respectively) and lower total body BMD (OR = 0.8687 in GTEx; 0.8919 in eQTLGen; *p* = 1.66E − 05 and 1.41E − 05, respectively). Age‐stratified analyses indicated that the association between mTOR expression and total body BMD varied by age. A significant inverse association was observed in individuals aged > 60 years (OR = 0.8275 in GTEx; 0.8708 in eQTLGen; *p* = 5.46E − 04 and 2.05E − 03), 45–60 years (OR = 0.8499 in GTEx; 0.8644 in eQTLGen; *p* = 5.38E − 03 and 3.44E − 03), and 30–45 years (OR = 0.8131 in GTEx; 0.8203 in eQTLGen; *p* = 9.51E − 03 and 3.82E − 03). No significant association was observed among individuals younger than 30 years. The HEIDI test did not indicate evidence of heterogeneity due to linkage (*p* > 0.01), supporting a shared causal variant rather than confounding by linkage disequilibrium. Consistent findings across both eQTLGen and GTEx datasets further strengthened the robustness of these results.

FIGURE 2(a) Forest plot showing the effect of genetically proxied mTOR expression on BMD (per SD increase). (b) Locus compare plot for the coloc test of SNVs associated with genetically proxied mTOR expression and heel BMD. Each point represented an SNV whose color indicated LD (r2).

**Figure:**
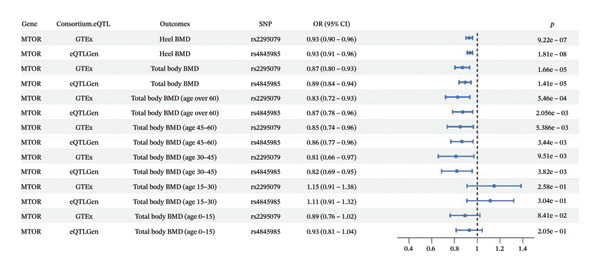
(a)

**Figure:**
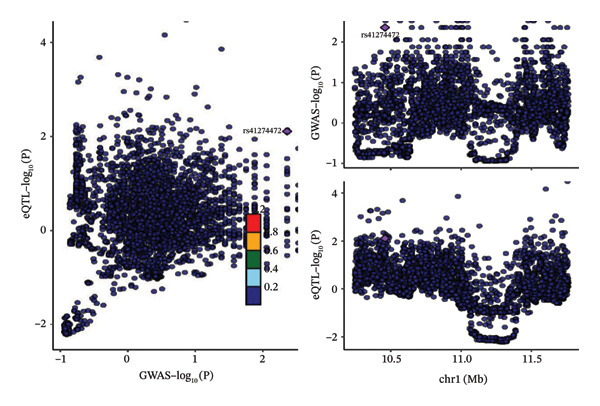
(b)

### 3.2. Evidence Suggestive of Colocalization Between Genetically Proxied mTOR Expression and Heel BMD

As a complementary analysis, Bayesian colocalization was performed to further evaluate whether the observed SMR association might be attributable to a shared causal variant, thereby reducing the likelihood of false‐positive findings. The coloc analysis (Figure 2(b); Supporting Tables 4 and 5) indicated suggestive evidence of shared genetic variation between mTOR expression and heel BMD (PP.H4 = 77.7%).

Overall, these findings provide genetic evidence consistent with a potential role of mTOR‐related pathways in BMD variation; however, they do not establish the clinical efficacy of pharmacological mTOR inhibition for osteoporosis.

### 3.3. Genetically Proxied mTOR Was Associated With Energy Metabolism–Related Phenotypes

As shown in Supporting Table 6, based on PheWAS results from GeneATLAS, we evaluated the associations between rs4845985 and 778 phenotypes. The volcano plot (Figure 3(a)) demonstrated that rs4845985 was primarily associated with body weight and limb impedance measures rather than BMD‐related traits, reducing the likelihood that the observed BMD associations were driven by horizontal pleiotropy. Notably, rs4845985 showed a strong association with basal metabolic rate (*β* = −16.769, FDR = 2.21E − 25) (Supporting Table 6), suggesting a potential link between mTOR‐related genetic variation and systemic energy metabolism.

FIGURE 3(a) PheWAS result for rs4845985. (b) Metabolic traits associated with genetically predicted mTOR expression. (c) Forest plot showing associations between mTOR‐associated metabolic traits and whole‐body bone mineral density across age groups. (d) MVMR forest plot for people over 60 years old. (e) MVMR forest plot for people aged 45–60 years old. (f) Boxplot of genetically predicted mTOR expression in blood.

**Figure:**
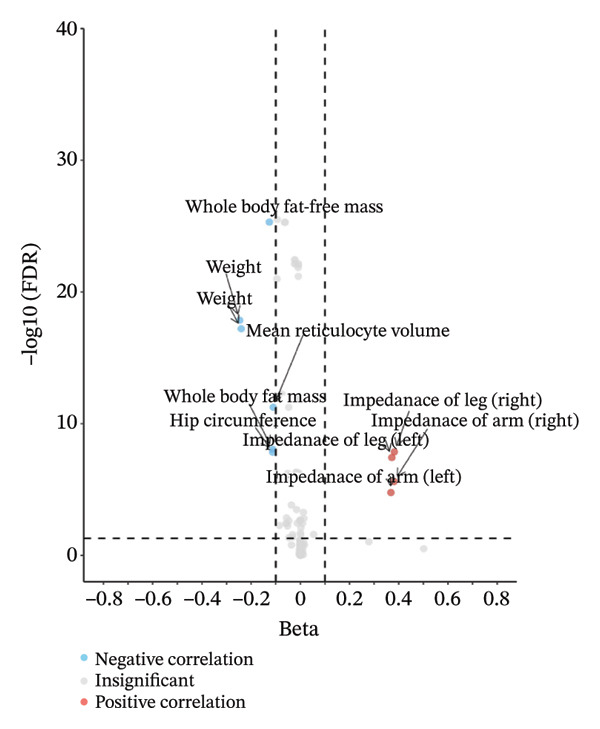
(a)

**Figure:**
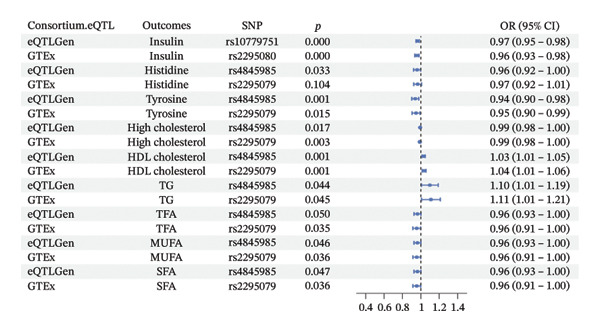
(b)

**Figure:**
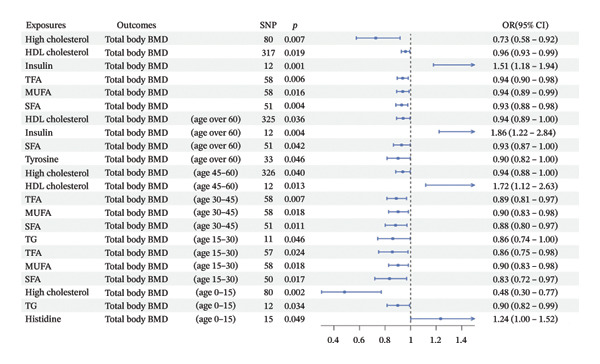
(c)

**Figure:**
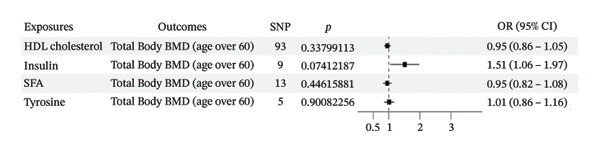
(d)

**Figure:**
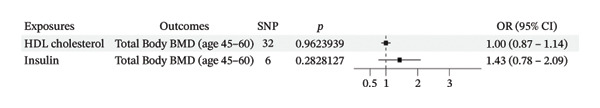
(e)

**Figure:**
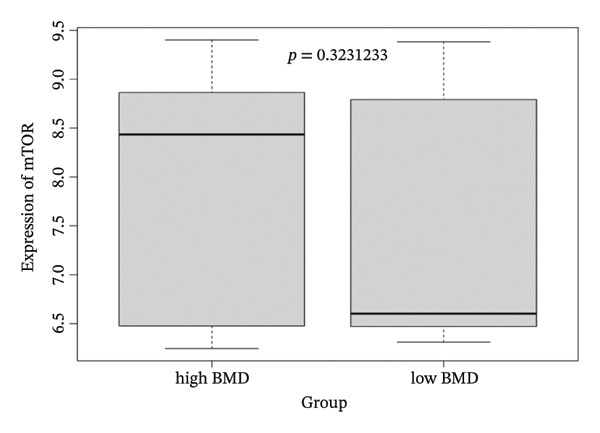
(f)

We examined the relationship between genetically predicted mTOR and several common markers related to energy metabolism using the SMR method, which included several hormones (fasting insulin, cortisol, thyroid‐stimulating hormone, leptin, adiponectin, and resistin), several indicators related to blood glucose (glucose, HbA1c, lactate, pyruvate, and HOMA‐IR score), blood amino acids (leucine, glycine, histidine, valine, alanine, tyrosine, glutamine, phenylalanine, concentration of branched‐chain amino acids [BCCAs]), blood lipids and cholesterol (diagnosed high cholesterol, HDL cholesterol, apolipoprotein A1, apolipoprotein B, LDL cholesterol, oxidized LDL receptor 1, VLDL cholesterol, and total triglycerides [TGs]), and blood fatty acids (total fatty acids [TFAs], monounsaturated fatty acids [MUFAs], polyunsaturated fatty acids [PUFAs], saturated fatty acids [SFAs], omega‐3 fatty acids, omega‐6 fatty acids, ratio of omega‐6 fatty acids to TFAs, and ratio of omega‐3 fatty acids to TFAs).

SMR analyses (Supporting Table 7; Figure 3(b)) indicated that per SD increase in genetically predicted mTOR expression, fasting insulin levels were lower (OR = 0.9571 in GTEx; 0.9660 in eQTLGen), tyrosine levels were lower (OR = 0.9480 in GTEx; 0.9392 in eQTLGen), and the odds of hypercholesterolemia were reduced (OR = 0.9891 in GTEx; 0.9925 in eQTLGen). HDL cholesterol levels were higher (OR = 1.0351 in GTEx; 1.0300 in eQTLGen), triglyceride levels were higher (OR = 1.1097 in GTEx; 1.0980 in eQTLGen), and TFA, MUFA, and SFA levels were lower in both datasets. Associations with histidine were directionally consistent but did not reach statistical significance. Overall, these findings indicate that genetically predicted mTOR expression is associated with multiple components of systemic energy metabolism, supporting a potential metabolic pathway underlying its relationship with BMD.

### 3.4. Age‐Stratified Associations Between mTOR‐Related Metabolic Traits and Systemic BMD

To evaluate whether metabolic traits associated with mTOR expression were related to BMD, we conducted two‐sample MR using the IVW method, followed by MVMR analyses. IVW‐MR results indicated that associations between metabolic traits and total body BMD varied across exposures and age strata (Table 1; Figure 3(c)). Genetically predicted higher insulin levels were positively associated with total body BMD (OR = 1.5129, *p* = 0.0011), including in individuals aged > 60 years (OR = 1.8642, *p* = 0.0037) and 45–60 years (OR = 1.7153, *p* = 0.0134). In contrast, genetically predicted higher levels of high cholesterol, HDL cholesterol, TFAs, MUFAs, SFAs, and tyrosine were inversely associated with BMD in specific age groups. Among individuals aged > 60 years, HDL cholesterol (OR = 0.9417, *p* = 0.0360), SFAs (OR = 0.9291, *p* = 0.0412), and tyrosine (OR = 0.9022, *p* = 0.0458) showed significant inverse associations with total body BMD. In those aged 45–60 years, HDL cholesterol remained inversely associated with BMD (OR = 0.9385, *p* = 0.0398). In individuals aged 30–45 years, higher TFAs (OR = 0.8880, *p* = 0.0071), MUFAs (OR = 0.9020, *p* = 0.0180), and SFAs (OR = 0.8823, *p* = 0.0106) were associated with lower systemic BMD. MR‐Egger analysis did not indicate directional pleiotropy, although heterogeneity was observed in some models (Table 1). For outcomes with evidence of heterogeneity, random‐effects IVW estimates were applied. All SNPs included in the IVW analyses are presented in Supporting Tables 8–13, and no weak IVs were identified (F‐statistics > 10). MVMR analyses were subsequently performed to estimate independent associations in individuals aged > 60 and 45–60 years. After jointly modeling the relevant metabolic traits, the inverse associations of HDL cholesterol, SFA, and tyrosine with BMD were attenuated and no longer statistically significant, whereas the positive association with insulin remained significant in individuals aged > 60 years (Figure 3(d)). In the 45–60‐year age group, none of the associations remained significant after multivariable adjustment (Figure 3(e)). Overall, these results indicate age‐specific differences in the associations between mTOR‐related metabolic traits and BMD, with insulin showing a more consistent positive association in older individuals and fatty acid–related traits demonstrating stronger inverse associations in younger adults.

**TABLE 1:** Associations of metabolic traits with total body BMD and sensitivity analyses.

**Outcomes**	**Exposures**	**SNPs**	**Inverse variance weighted (IVW)**			**MR-Egger**	**Weighted**	**Simple**	**Weighted**	**Heterogeneity**		**Pleiotropy (MR-Egger)**
							**Median**	**Mode**	**Mode**	**(*Q*-value)**		**(** **p** **value)**
			**b (se)**	**p** **value**	**OR (95% CI**	**b (se)**	**b (se)**	**b (se)**	**b (se)**	**IVW**	**MR-Egger**	
Total body BMD	High cholesterol	80	−0.318 (0.12)	**0.0073**	**0.7273 (0.5763, 0.9178)**	−0.568 (0.12)	−0.221 (0.15)	−0.460 (0.28)	−0.304 (0.16)	0.0014	0.0021	0.1511
	HDL cholesterol	317	−0.041 (0.02)	**0.0186**	**0.9599 (0.9277, 0.9932)**	−0.020 (0.03)	−0.005 (0.03)	−0.069 (0.06)	−0.010 (0.02)	7.35E − 09	7.91E − 09	0.2996
	Insulin	12	0.414 (0.13)	**0.0011**	**1.5129 (1.1792, 1.9410)**	1.584 (0.66)	0.467 (0.03)	0.033 (0.32)	0.654 (0.28)	0.2739	0.4389	0.1002
	TG	11	−0.033 (0.02)	0.1584	0.9777 (0.9223, 1.0364)	−0.008 (0.06)	−0.044 (0.03)	−0.054 (0.05)	−0.051 (0.03)	0.1992	0.1533	0.6905
	TFA	58	−0.064 (0.02)	**0.0060**	**0.9376 (0.8954, 0.9817)**	−0.081 (0.04)	−0.053 (0.03)	0.018 (0.06)	−0.034 (0.03)	6.23E − 05	5.01E − 05	0.6091
	MUFA	58	−0.064 (0.02)	**0.0163**	**0.9383 (0.8908, 0.9884)**	−0.069 (0.04)	−0.051 (0.03)	−0.070 (0.05)	−0.036 (0.03)	6.84E − 09	4.35E − 09	0.8693
	SFA	51	−0.073 (0.03)	**0.0045**	**0.9297 (0.8841, 0.9776)**	−0.076 (0.05)	−0.070 (0.03)	−0.006 (0.06)	−0.038 (0.04)	0.0004	0.0002	0.9362
	Histidine	14	0.136 (0.07)	0.0578	1.1456 (0.9955, 1.3183)	0.051 (0.20)	0.143 (0.07)	0.085 (0.07)	0.138 (0.06)	0.0003	0.0002	0.6580
	Tyrosine	32	−0.050 (0.04)	0.2298	0.9508 (0.8757, 1.0324)	−0.005 (0.07)	−0.037 (0.04)	−0.110 (0.08)	0.001 (0.03)	8.39E − 07	9.60E − 07	0.3994

Total body BMD (age over 60)	High cholesterol	81	−0.286 (0.19)	0.1242	0.7512 (0.5216, 1.0818)	−0.479 (0.33)	−0.353 (0.24)	−0.523 (0.46)	−0.358 (0.25)	0.0219	0.0203	0.4825
	HDL cholesterol	325	−0.060 (0.03)	**0.0360**	**0.9417 (0.8903, 0.9961)**	−0.023 (0.04)	−0.031 (0.05)	−0.229 (0.11)	−0.009 (0.04)	2.77E − 07	3.00E − 07	0.2810
	Insulin	12	0.622 (0.21)	**0.0037**	**1.8642 (1.2235, 2.8404)**	1.700 (1.17)	0.868 (0.26)	1.007 (0.44)	1.060 (0.41)	0.1286	0.1301	0.3683
	TG	12	−0.051 (0.04)	0.2391	0.9505 (0.8734, 1.0343)	0.076 (0.12)	−0.021 (0.05)	0.045 (0.08)	0.015 (0.06)	0.0477	0.0630	0.2810
	TFA	58	−0.054 (0.03)	0.0906	0.9470 (0.8892, 1.0087)	−0.059 (0.06)	−0.044 (0.05)	−0.042 (0.11)	−0.053 (0.06)	0.1523	0.1320	0.9230
	MUFA	58	−0.053 (0.04)	0.1554	0.9483 (0.8812, 1.0203)	−0.051 (0.06)	−0.037 (0.05)	−0.065 (0.10)	−0.072 (0.05)	0.0015	0.0012	0.9707
	SFA	51	−0.074 (0.04)	**0.0412**	**0.9291 (0.8656, 0.9973)**	−0.074 (0.04)	−0, 044 (0.05)	−0.057 (0.12)	−0.063 (0.07)	0.1435	0.1421	0.3604
	Histidine	15	0.083 (0.07)	0.2437	1.0860 (0.9454, 1.2476)	0.154 (0.18)	0.181 (0.10)	0.127 (0.16)	0.164 (0.11)	0.5738	0.5090	0.6819
	Tyrosine	33	−0.103 (0.05)	**0.0458**	**0.9022 (0.8155, 0.9981)**	−0.020 (0.08)	−0.012 (0.05)	−0.137 (0.13)	−0.009 (0.06)	0.0741	0.0905	0.2081

Total body BMD (age 45−60)	High cholesterol	80	−0.196 (0.22)	0.3617	0.8217 (0.5389, 1.2530)	−0.728 (0.38)	−0.356 (0.27)	−0.232 (0.52)	−0.405 (0.27)	0.0088	0.0140	0.0945
	HDL cholesterol	326	−0.063 (0.03)	**0.0398**	**0.9385 (0.8834, 0.9970)**	−0.014 (0.05)	−0.027 (0.05)	−0.075 (0.10)	−0.022 (0.04)	4.05E − 06	5.01E − 06	0.1703
	Insulin	12	0.540 (0.22)	**0.0134**	**1.7153 (1.1182, 2.6312)**	−0.354 (1.36)	0.496 (0.30)	0.500 (0.45)	0.493 (0.43)	0.9380	0.9149	0.5213
	TG	12	−0.030 (0.05)	0.5805	0.9701 (0.8712, 1.0803)	0.126 (0.15)	0.017 (0.05)	−0.068 (0.08)	−0.003 (0.06)	0.0047	0.0074	0.3028
	TFA	58	−0.042 (0.04)	0.2677	0.9588 (0.8900, 1.0329)	−0.030 (0.07)	0.030 (0.05)	0.008 (0.09)	0.032 (0.06)	0.0256	0.0210	0.8194
	MUFA	58	−0.050 (0.04)	0.2280	0.9508 (0.8759, 1.0320)	−0.053 (0.07)	0.024 (0.05)	−0.022 (0.10)	0.016 (0.05)	0.0004	0.0003	0.9655
	SFA	51	−0.045 (0.04)	0.2800	0.9564 (0.8830, 1.0360)	−0.036 (0.07)	0.035 (0.06)	0.022 (0.11)	0.062 (0.07)	0.0760	0.0635	0.8844
	Histidine	15	0.159 (0.11)	0.1348	1.1728 (0.9517, 1.4453)	−0.204 (0.27)	0.127 (0.11)	0.112 (0.17)	0.118 (0.12)	0.0251	0.0500	0.1654
	Tyrosine	33	−0.081 (0.06)	0.1424	0.9221 (0.8275, 1.0276)	−0.070 (0.09)	−0.032 (0.07)	−0.124 (0.13)	−0.034 (0.06)	0.0713	0.0568	0.8787

Total body BMD (age 30−45)	High cholesterol	80	−0.359 (0.25)	0.1468	0.6986 (0.4304, 1.1340)	−0.019 (0.44)	−0.144 (0.38)	0.134 (0.76)	−0.049 (0.47)	0.7398	0.7371	0.3590
	HDL cholesterol	327	−0.056 (0.04)	0.8861	0.9943 (0.9201, 1.0746)	0.031 (0.06)	0.007 (0.07)	−0.127 (0.14)	0.009 (0.06)	0.0062	0.0060	0.4368
	Insulin	14	0.492 (0.31)	0.1184	1.6349 (0.8821, 3.0303)	2.413 (1.69)	0.561 (0.39)	0.379 (0.71)	0.729 (0.58)	0.1700	0.1952	0.2710
	TG	12	−0.063 (0.05)	0.1787	0.9368 (0.8517, 1.0303)	−0.021 (0.14)	−0.044 (0.07)	−0.075 (0.11)	−0.031 (0.08)	0.5242	0.4451	0.7347
	TFA	58	−0.119 (0.04)	**0.0071**	**0.8880 (0.8144, 0.9682)**	−0.131 (0.08)	−0.100 (0.06)	−0.129 (0.13)	−0.106 (0.08)	0.9591	0.9500	0.8423
	MUFA	58	−0.103 (0.04)	**0.0180**	**0.9020 (0.8281, 0.9825)**	−0.081 (0.07)	−0.026 (0.07)	−0.112 (0.11)	−0.062 (0.07)	0.6795	0.6503	0.6936
	SFA	51	−0.125 (0.05)	**0.0106**	**0.8823 (0.8015, 0.9713)**	−0.173 (0.09)	−0.128 (0.07)	−0.157 (0.14)	−0.145 (0.09)	0.9881	0.9867	0.5031
	Histidine	15	0.133 (0.11)	0.2163	1.1417 (0.9254, 1.4086)	0.007 (0.28)	0.056 (0.15)	−0.072 (0.25)	0.030 (0.19)	0.5198	0.4596	0.6340
	Tyrosine	33	0.021 (0.07)	0.7686	1.0219 (0.8846, 1.1804)	0.029 (0.12)	0.051 (0.10)	−0.080 (0.20)	−0.025 (0.10)	0.1231	0.1002	0.9384
Total body BMD (age 15−30)	High cholesterol	78	−0.246 (0.41)	0.5493	0.7821 (0.3499, 1.7484)	−1.045 (0.74)	−0.288 (0.61)	−0.234 (1.17)	−0.480 (0.79)	0.1802	0.1970	0.1984
	HDL cholesterol	324	−0.031 (0.06)	0.5754	0.9693 (0.8693, 1.0809)	−0.038 (0.09)	−0.150 (0.10)	−0.219 (0.20)	−0.118 (0.09)	0.9895	0.9883	0.9138
	Insulin	14	0.431 (0.50)	0.3843	1.5390 (0.5826, 4.0652)	5.035 (2.42)	0.659 (0.59)	0.8880 (1.25)	0.720 (1.16)	0.1384	0.2929	0.0769
	TG	11	−0.150 (0.08)	**0.0463**	**0.8606 (0.7425, 0.9975)**	−0.068 (0.21)	−0.145 (0.10)	−0.133 (0.16)	−0.141 (0.14)	0.6070	0.5288	0.6898
	TFA	57	−0.154 (0.07)	**0.0240**	**0.8571 (0.7497, 0.9799)**	−0.252 (0.12)	−0.216 (0.11)	−0.072 (0.20)	−0.293 (0.11)	0.5900	0.5927	0.3106
	MUFA	58	−0.103 (0.04)	**0.0181**	**0.9020 (0.8281, 0.9825)**	−0.081 (0.07)	−0.026 (0.07)	−0.112 (0.11)	−0.062 (0.07)	0.3500	0.3718	0.2131
	SFA	50	−0.180 (0.08)	**0.0174**	**0.8349 (0.7196, 0.9688)**	−0.272 (0.13)	−0.245 (0.11)	−0.012 (0.23)	−0.320 (0.12)	0.8737	0.8693	0.4044
	Histidine	15	0.324 (0.17)	0.0545	1.3821 (0.9937, 1.9222)	0.408 (0.44)	0.180 (0.22)	0.236 (0.36)	0.169 (0.28)	0.8642	0.8157	0.8377
	Tyrosine	33	0.003 (0.10)	0.9789	1.0027 (0.8196, 1.2269)	0.079 (0.17)	0.079 (0.15)	0.081 (0.27)	0.117 (0.16)	0.6570	0.6256	0.5671

Total body BMD (age 0−15)	High cholesterol	80	−0.726 (0.24)	0.0022	0.4839 (0.3041, 0.7702)	−0.838 (0.42)	−0.600 (0.38)	−0.484 (0.84)	−0.484 (0.54)	0.0863	0.0760	0.7198
	HDL cholesterol	327	−0.000 (0.03)	0.9939	0.9997 (0.9376, 1.0661)	−0.032 (0.05)	−0.010 (0.06)	−0.127 (0.11)	−0.023 (0.05)	0.1600	0.1570	0.4202
	Insulin	14	0.311 (0.24)	0.1940	1.3645 (0.8537, 2.1809)	2.005 (1.30)	0.190 (0.32)	0.188 (0.56)	0.156 (0.53)	0.7070	0.7783	0.2101
	TG	12	−0.106 (0.05)	0.0342	0.8994 (0.8155, 0.9921)	−0.267 (0.14)	−0.134 (0.06)	−0.141 (0.10)	−0.149 (0.06)	0.1685	0.2109	0.2382
	TFA	57	−0.042 (0.05)	0.4040	0.9590 (0.8691, 1.0581)	−0.111 (0.09)	−0.020 (0.06)	0.040 (0.14)	−0.063 (0.07)	0.0013	0.0014	0.3318
	MUFA	57	−0.058 (0.04)	0.1910	0.9435 (0.8648, 1.0294)	−0.062 (0.07)	−0.128 (0.06)	0.114 (0.13)	−0.116 (0.07)	0.047	0.0389	0.9416
	SFA	50	−0.041 (0.06)	0.4759	0.9601 (0.8586, 1.0738)	−0.105 (0.10)	−0.007 (0.07)	0.108 (0.14)	−0.031 (0.08)	0.0010	0.0010	0.4462
	Histidine	15	0.211 (0.11)	0.0489	1.2353 (1.0010, 1.5243)	0.389 (0.28)	0.336 (0.13)	0.410 (0.18)	0.360 (0.15)	0.2163	0.1909	0.5097
	Tyrosine	33	0.018 (0.07)	0.8174	1.0179 (0.8764, 1.1820)	0.051 (0.12)	0.105 (0.08)	−0.006 (0.16)	0.076 (0.08)	0.0048	0.0036	0.7382

*Note:* Bold values indicates statistically significant differences (*p* < 0.05).

### 3.5. No Difference in Peripheral Blood mTOR Expression Between High and Low BMD Groups

We further examined mTOR transcription levels in blood monocytes among Caucasian women stratified by high and low BMD. As shown in Figure 3(f), no statistically significant difference in mTOR expression was observed between the two groups (*p* = 0.3231). These findings indicate that peripheral blood mTOR expression levels do not differ between individuals with high and low BMD in this cohort, suggesting that baseline transcriptional differences in blood monocytes may not account for the observed genetic associations (Figure 3(f)).

### 3.6. Molecular Docking Analysis of mTOR and Fatty Acids

Given that our MR analyses suggested that circulating fatty acid levels may be associated with mTOR‐related effects on BMD, we further explored whether fatty acids could directly interact with mTOR at the molecular level. Fatty acids were selected as candidate ligands because they are key metabolic substrates closely involved in energy metabolism and bone homeostasis. We therefore performed molecular docking analyses to evaluate the potential binding interactions between mTOR and several common fatty acids and calculated their predicted binding energies (Supporting Table 14). The selected ligands included the saturated fatty acids palmitic acid (C16:0) and stearic acid (C18:0), the monounsaturated fatty acid oleic acid (C18:1), and the polyunsaturated fatty acids linoleic acid (C18:2) and arachidonic acid (C20:4).

Among the tested ligands, arachidonic acid exhibited the strongest predicted binding affinity (−5.9 kcal/mol), followed by palmitic acid (−5.0 kcal/mol), oleic acid (−4.9 kcal/mol), linoleic acid (−4.8 kcal/mol), and stearic acid (−4.4 kcal/mol). The predicted docking conformations indicated that these fatty acids may interact with several key residues of mTOR through hydrogen bonding and hydrophobic interactions (Figure 4). As an exploratory analysis, these findings suggest a potential direct interaction between fatty acids and mTOR, which may provide a mechanistic hypothesis underlying the epidemiological associations observed in our MR analyses.

FIGURE 4Molecular docking interactions between mTOR and five representative fatty acids: (a) stearic acid, (b) palmitic acid, (c) oleic acid, (d) linoleic acid, and (e) arachidonic acid.

**Figure:**
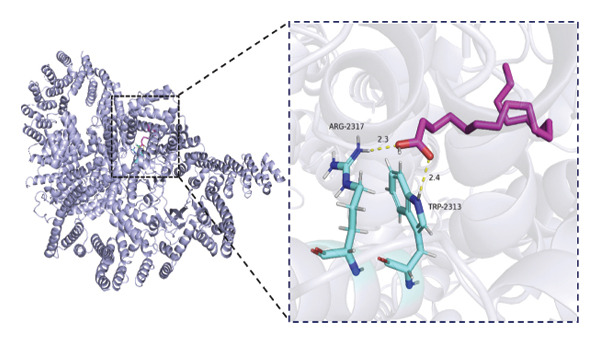
(a)

**Figure:**
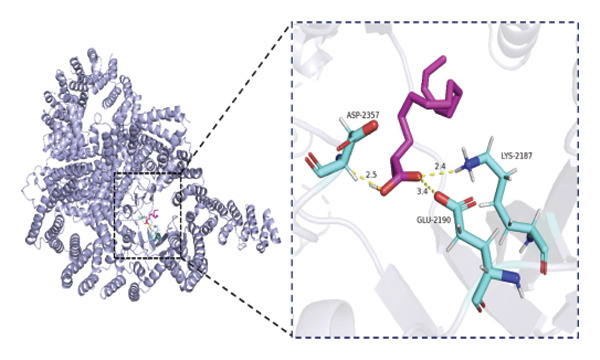
(b)

**Figure:**
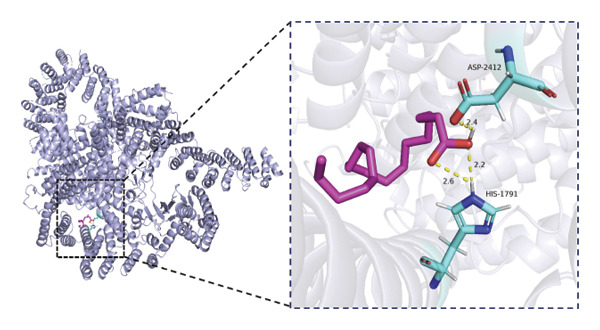
(c)

**Figure:**
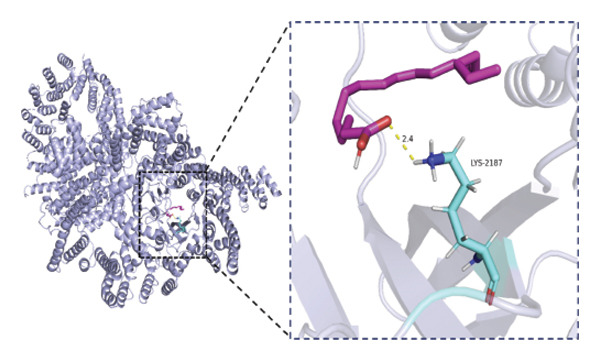
(d)

**Figure:**
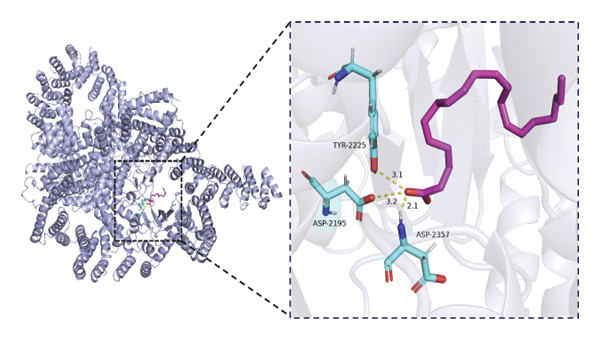
(e)

## 4. Discussion

This is, to our knowledge, the first genetics‐based study to investigate whether mTOR‐related targets are associated with BMD. Our findings suggest that a genetically predicted lower mTOR expression in blood is associated with higher BMD, with evidence consistent with colocalization between mTOR expression and BMD signals. These results are in line with previous experimental and observational studies [6–9]. Notably, the association appeared to be age‐dependent: genetically predicted lower mTOR expression was not significantly associated with BMD among individuals under 30 years of age. To our knowledge, no prior human genetic study has reported this age‐specific pattern. We speculate that aging biology, musculoskeletal homeostasis, and the mTOR signaling pathway may share overlapping mechanisms, which could partly explain the observed age‐related differences [32].

The biological mechanisms underlying the potential effects of mTOR inhibition on osteoporosis remain under investigation. Previous experimental studies provide important mechanistic insights. One possible explanation may relate to the well‐documented antiaging properties of mTOR inhibitors [32]. Aging is a major risk factor for osteoporosis, and the majority of affected individuals are older adults [1]. Over the past 2 decades, rapamycin has been shown to extend lifespan in multiple model organisms, including mice, *Drosophila*, and yeast [33]. Inhibition of mTOR signaling has also been implicated in the modulation of age‐related diseases. For example, rapamycin has been reported to attenuate dopaminergic neuron loss in *Drosophila* models and in mouse models of Alzheimer’s disease [34]. mTOR plays a central role in the regulation of cellular autophagy, a lysosome‐mediated process responsible for the degradation of damaged organelles and macromolecules [35]. Autophagy is involved in numerous physiological and pathological processes and is highly relevant in pharmacological research [35]. Activation and recruitment of mTORC1 (mTOR Complex 1) are critical for lysosomal regulation and have been shown to suppress autophagy through phosphorylation of autophagy‐related proteins, including ULK1, FIP200, and ATG13, thereby inhibiting formation of the ULK1 complex [36]. In addition, accumulating evidence suggests that multiple small‐molecule compounds may influence bone density through mTOR‐dependent pathways [37–39]. Our findings are broadly consistent with these experimental observations, supporting a potential role of mTOR signaling in bone metabolism.

Equally, the metabolic effects associated with mTOR inhibition warrant careful evaluation prior to any clinical consideration. In oncology and other therapeutic contexts, mTOR inhibitors have been reported to influence energy and nutrient metabolism. For example, Bouillet B et al. [40] reported that patients receiving mTOR inhibitors as anticancer therapy frequently developed hyperglycemia (12%–50%) and hyperlipidemia (7%–73%). Similarly, Mulder FVM et al. [41] observed that long‐term administration of mTOR inhibitors in patients with tuberous sclerosis was associated with hypercholesterolemia (20%–25% during the first year), whereas the prevalence of diabetes mellitus was relatively low (2.5%). Due to differences in study design, endpoints, and patient populations, these findings are not entirely consistent, but collectively they suggest that mTOR modulation has broad metabolic consequences. Importantly, it should be emphasized that MR estimates the effect of lifelong genetically predicted variation in gene expression, which is not equivalent to short‐term pharmacological inhibition. Therefore, our findings cannot be directly interpreted as predicting the clinical efficacy or adverse‐effect profile of mTOR inhibitors. Rather, they reflect the consequences of genetically proxied differences in mTOR expression over the life course. In our analyses, genetically predicted insulin levels appeared to attenuate certain mTOR‐associated metabolic signals in individuals over 45 years of age. However, this observation should be interpreted cautiously. The suggestion that insulin regulation may counteract mTOR‐related metabolic perturbations represents a hypothesis based on statistical association rather than direct mechanistic evidence. One possible explanation is that altered mTOR expression could influence the insulin/IGF‐1–signaling pathway [7], thereby modifying downstream metabolic phenotypes. Our phenome‐wide analyses also align with emerging genetic evidence linking mTOR‐related variation to metabolic traits [42]. Studies have reported that genetically predicted mTOR expression was associated with body mass index and related metabolic phenotypes. These findings complement our results, reinforcing the notion that mTOR signaling is broadly implicated in metabolic regulation. However, together these studies underscore that mTOR‐related genetic effects extend beyond bone metabolism, highlighting the need for cautious interpretation when considering potential therapeutic implications. Nevertheless, this remains speculative and requires experimental validation.

Our study has several strengths. As a genetics‐based drug target investigation, it reduces confounding and reverse causation compared with conventional observational studies. Multiple independent cohorts were used for validation, and consistent results were observed across datasets. Sensitivity analyses were conducted in accordance with the core assumptions of MR.

However, several limitations should be acknowledged. First, our use of blood mTOR expression as a genetic proxy does not capture tissue‐specific effects. The absence of differential mTOR transcription in peripheral blood between high and low BMD groups should not be interpreted as evidence of therapeutic neutrality. Rather, it may reflect tissue specificity, as mTOR activity in bone, liver, pancreas, or immune cells could differ substantially from that in circulating blood cells. This limitation should be considered when interpreting the translational implications of our findings. Second, our cohorts were predominantly of European ancestry, which may limit generalizability to other populations. Although some GWAS meta‐analyses included participants of diverse backgrounds, potential residual population structure cannot be excluded. In addition, the use of independent datasets to minimize sample overlap may have introduced heterogeneity across analyses. Third, potential mediators related to nutritional metabolism and endocrine function—such as insulin resistance, baseline metabolic disease, or other hormonal factors—were not directly modeled. These factors may influence the relationship between mTOR signaling and osteoporosis and warrant further investigation. Finally, experimental data regarding rapamycin and bone remain inconsistent. One study reported that short‐term, low‐dose rapamycin had no significant effect on bone quality in young or old mice [43], whereas another found that low‐dose rapamycin promoted osteoclastogenesis and bone loss [44]. These discrepancies suggest that the skeletal effects of mTOR inhibition may depend on dose, duration, and physiological context. Moreover, rapamycin’s immunosuppressive actions are mediated through mTORC1 in immune cells, and our blood‐based genetic proxy does not directly assess immune functional changes. Therefore, any clinical translation would require careful evaluation of both skeletal and systemic effects.

In summary, although our findings support a potential association between mTOR signaling and bone metabolism, they should not be interpreted as direct evidence for pharmacological intervention. Any clinical application would require rigorous experimental validation and careful consideration of the balance between potential skeletal benefits and known systemic risks. Future studies integrating multiomics data, multicenter cohorts, and diverse populations may further clarify the role of mTOR in osteoporosis and systemic metabolism.

## 5. Conclusions

Our study comprehensively evaluated the potential efficacy and side effects of mTOR inhibitors on osteoporosis. Our findings could provide a theoretical basis for osteoporosis drug development and avoid the failure rate and incidence of adverse effects in future clinical trials.

**Nomenclature**
mTOR: Mammalian target of rapamycin
GWAS: Genome‐wide association study
eQTL: Expression quantitative trait locus
GEO: Gene Expression Omnibus
BMD: Bone mineral density
MR: Mendelian randomization
MVMR: Multivariate Mendelian randomization
SMR: Summary data–based Mendelian randomization
SNP: Single‐nucleotide polymorphism
SNV: Single‐nucleotide variant
IVW: Inverse variance weighted
IV: Instrumental variable
PheWAS: Phenome‐wide association study
MAF: Minor allele frequency
mTORC1: mTOR Complex 1

## Author Contributions

ZhaoLiang Zhang and LieHui Yao conceptualized the study and drafted the manuscript. Tao Ma conducted data analysis and visualization. LiMing Zhou supervised the study, provided overall guidance, and reviewed the final manuscript. YunFei Xu participated in the literature review and interpretation of results and serves as the corresponding author. He led the revision process, addressed reviewers’ comments, coordinated manuscript modifications, and finalized the revised submission.

## Funding

This study was supported by a grant from the Yixing People’s Hospital (Project No. YRY2025B002) and Wuxi Municipal Health Commission (Project No. Q202568).

## Ethics Statement

The studies involving humans were approved by the relevant ethics committees. This study was conducted in accordance with the Declaration of Helsinki. All cited GWAS and other data have been approved by the relevant review boards. The studies were conducted in accordance with the local legislation and institutional requirements. The human samples used in this study were obtained from open‐access original studies and corresponding databases. All original research were derived from open‐access publications and the corresponding databases. Written informed consent for participation was not required from the participants or the participants’ legal guardians/next of kin in accordance with the national legislation and institutional requirements.

## Consent

All authors consent to publication.

## Conflicts of Interest

The authors declare no conflicts of interest.

## Supporting Information

Additional supporting information can be found online in the Supporting Information section.

## Supporting information


**Supporting Information** Supporting table 1: Baseline information for cited studies. Supporting table 2: SMR analysis and HEIDI test for genetically proxied mTOR and BMD. Supporting table 3: Information about genetic instrumental variables for mTOR (*Cis*‐eQTLs). Supporting table 4: Bayesian colocalization results for mTOR instrumental variables. Supporting table 5: Bayesian colocalization results for mTOR with heel BMD. Supporting table 6: PheWAS results of the top SNP (rs4845985) of mTOR. Supporting table 7: SMR analysis and HEIDI test for mTOR and energy metabolism–related traits. Supporting table 8: Genetic instrumental variables for mTOR‐related metabolic traits and total body BMD. Supporting table 9: Genetic instrumental variables for mTOR‐related metabolic traits and total body BMD (age over 60). Supporting table 10: Genetic instrumental variables for mTOR‐related metabolic traits and total body BMD (age 45–60). Supporting table 11: Genetic instrumental variables for mTOR‐related metabolic traits and total body BMD (age 30–45). Supporting table 12: Genetic instrumental variables for mTOR‐related metabolic traits and total body BMD (age 15–30). Supporting table 13: Genetic instrumental variables for mTOR‐related metabolic traits and total body BMD (age 0–15). Supporting table 14: Molecular binding energies between mTOR and five fatty acids. Supporting figure 1: Expression boxplots of GSE56814 and GSE56815 before (a) and after (b) the removal of batch effects. PCA plots for high BMD and low BMD groups (c). PCA plot of GSE56814 and GSE56815 (d). Supporting list: STROBE‐MR checklist for the study.

## Data Availability

The datasets presented in this study can be found in online repositories. The names of the repository/repositories and accession number(s) can be found below: All sources of publicly available data are available open source in relevant studies (Supporting table 1). GWAS summary‐level data were obtained from the IEU database (https://gwas.mrcieu.ac.uk/datasets/). EQTL data are available at the eQTLGen Consortium (https://www.eqtlgen.org/) and GTEx Consortium V8 (https://gtexportal.org/). Transcriptome datasets are available in the GEO database (https://www.ncbi.nlm.nih.gov/geo/): GSE56814 and GSE56815. The three‐dimensional structures of ligands were downloaded from PubChem (https://pubchem.ncbi.nlm.nih.gov/), and the crystal structure of mTOR was obtained from the Protein Data Bank (https://www.rcsb.org/). Docking was performed using AutoDock Vina 1.1.2, and results were visualized with PyMOL 2.6.0. Data for all individuals have been uploaded to Supporting information (Supporting tables 1–14 and Supporting figure 1).
