# Supplementary material for: Mendelian Randomization Revealed Potential of mTOR Inhibitors for Treatment of Osteoporosis: Evidence From GWAS and Transcriptome Data
Source: Int J Endocrinol. 2026 May 23;2026:5562148. doi: 10.1155/ije/5562148 (PMC13197831; doi:10.1155/ije/5562148)
Supplement: Supplementary file 1 — Supporting Information Supporting table 1: Baseline information for cited studies. Supporting table 2: SMR analysis and HEIDI test for genetically proxied mTOR and BMD. Supporting table 3: Information about genetic instrumental variables for mTOR (Cis‐eQTLs). Supporting table 4: Bayesian colocalization results for mTOR instrumental variables. Supporting table 5: Bayesian colocalization results for mTOR with heel BMD. Supporting table 6: PheWAS results of the top SNP (rs4845985) of mTOR. Supporting table 7: SMR analysis and HEIDI test for mTOR and energy metabolism–related traits. Supporting table 8: Genetic instrumental variables for mTOR‐related metabolic traits and total body BMD. Supporting table 9: Genetic instrumental variables for mTOR‐related metabolic traits and total body BMD (age over 60). Supporting table 10: Genetic instrumental variables for mTOR‐related metabolic traits and total body BMD (age 45–60). Supporting table 11: Genetic instrumental variables for mTOR‐related metabolic traits and total body BMD (age 30–45). Supporting table 12: Genetic instrumental variables for mTOR‐related metabolic traits and total body BMD (age 15–30). Supporting table 13: Genetic instrumental variables for mTOR‐related metabolic traits and total body BMD (age 0–15). Supporting table 14: Molecular binding energies between mTOR and five fatty acids. Supporting figure 1: Expression boxplots of GSE56814 and GSE56815 before (a) and after (b) the removal of batch effects. PCA plots for high BMD and low BMD groups (c). PCA plot of GSE56814 and GSE56815 (d). Supporting list: STROBE‐MR checklist for the study. [file IJE-2026-5562148-s001.zip › Supplementary list.docx]

STROBE Statement—checklist

|  | Item No. | Recommendation | | Relevant text from manuscript |
| --- | --- | --- | --- | --- |
| **Title and abstract** | 1 | (*a*) Indicate the study’s design with a commonly used term in the title or the abstract | | The title indicates that “Mendelian randomization revealed …”. |
|  |  | (*b*) Provide in the abstract an informative and balanced summary of what was done and what was found | | The background states that the study aims to use Mendelian randomization to elucidate the causal relationship between mTOR and BMD. |
|  | | |  |  |
| Background/rationale | 2 | Explain the scientific background and rationale for the investigation being reported | | Introduces mTOR and its potential role in osteoporosis; mentions pharmacological experiments and genetic approaches. Statements such as “Genetic approaches provide an alternative…reduce confounding” already describe the value of Mendelian randomization. |
| Objectives | 3 | State specific objectives, including any prespecified hypotheses | | The Aims section states the study hypothesis: “We hypothesized that…”. |
|  | | |  |  |
| Study design | 4 | Present key elements of study design early in the paper | | The Methods section of the manuscript states that a two-sample Mendelian randomization approach was used and briefly describes the sources of the datasets. |
| Setting | 5 | Describe the setting, locations, and relevant dates, including periods of recruitment, exposure, follow-up, and data collection | | Information on sample sources, cohort characteristics, and years of data collection for each dataset is presented in Supplementary Table 1. |
| Participants | 6 | (*a*) *Cohort study*—Give the eligibility criteria, and the sources and methods of selection of participants. Describe methods of follow-up  *Case-control study*—Give the eligibility criteria, and the sources and methods of case ascertainment and control selection. Give the rationale for the choice of cases and controls  *Cross-sectional study*—Give the eligibility criteria, and the sources and methods of selection of participants | | The study used publicly available GWAS and eQTL summary data, with no independent participant recruitment; all analyzed participants were primarily adults of European ancestry. |
|  |  | (*b*) *Cohort study*—For matched studies, give matching criteria and number of exposed and unexposed  *Case-control study*—For matched studies, give matching criteria and the number of controls per case | | Not appliable. |
| Variables | 7 | Clearly define all outcomes, exposures, predictors, potential confounders, and effect modifiers. Give diagnostic criteria, if applicable | | Exposure: genetically predicted mTOR expression; Outcome: body BMD; Potential confounders: controlled through the MR design, not directly measured. |
| Data sources/ measurement | 8* | For each variable of interest, give sources of data and details of methods of assessment (measurement). Describe comparability of assessment methods if there is more than one group | | The data sources are described in the Methods section, and the measurement details are consistent with the original studies.. |
| Bias | 9 | Describe any efforts to address potential sources of bias | | The main biases are horizontal pleiotropy and linkage disequilibrium. The study assessed pleiotropy using the HEIDI test, MR-Egger intercept, and MR-PRESSO. |
| Study size | 10 | Explain how the study size was arrived at | | Existing large-scale publicly available summary-level datasets were used, so no additional sample size calculation was required. |

Continued on next page

| Quantitative variables | 11 | Explain how quantitative variables were handled in the analyses. If applicable, describe which groupings were chosen and why |  | The Statistical Methods section describes how quantitative variables were handled and how groups were defined. |
| --- | --- | --- | --- | --- |
| Statistical methods | 12 | (*a*) Describe all statistical methods, including those used to control for confounding |  | The primary analysis used SMR software (v1.3.1) to estimate the association between mTOR expression and BMD, while IVW-MR and MVMR analyses were conducted using the TwoSampleMR R package (v0.5.6). No additional covariate adjustment was performed because summary-level data were used |
|  |  | (*b*) Describe any methods used to examine subgroups and interactions |  | Age-stratified IVW-MR analyses were performed to estimate effect sizes in different age groups. MVMR simultaneously included genetic instruments for mTOR and metabolic markers to assess the independent effects of each exposure. |
|  |  | (*c*) Explain how missing data were addressed |  | Summary-level data were used, and no missing data handling was reported. |
|  |  | (*d*) *Cohort study*—If applicable, explain how loss to follow-up was addressed  *Case-control study*—If applicable, explain how matching of cases and controls was addressed  *Cross-sectional study*—If applicable, describe analytical methods taking account of sampling strategy |  | Not appliable. |
|  |  | (*e*) Describe any sensitivity analyses |  | HEIDI tests, multiple testing correction, and MR-PRESSO were performed to account for pleiotropy, and all sensitivity analysis results should be reported in the Results section. |
| Results | | | | |
| Participants | 13* | (a) Report numbers of individuals at each stage of study—eg numbers potentially eligible, examined for eligibility, confirmed eligible, included in the study, completing follow-up, and analysed |  | Publicly available summary-level data were used, with no individual-level participant workflow reported. |
|  |  | (b) Give reasons for non-participation at each stage |  |  |
|  |  | (c) Consider use of a flow diagram |  |  |
| Descriptive data | 14* | (a) Give characteristics of study participants (eg demographic, clinical, social) and information on exposures and potential confounders |  | The UKBB heel BMD data were reported (mean = 0.544 g/cm², SD = 0.139), but participant characteristics were not systematically listed, and missing data were not addressed. |
|  |  | (b) Indicate number of participants with missing data for each variable of interest |  |  |
|  |  | (c) *Cohort study*—Summarise follow-up time (eg, average and total amount) |  |  |
| Outcome data | 15* | *Cohort study*—Report numbers of outcome events or summary measures over time |  | This study used continuous BMD values, and the association estimates were reported per SD unit in the Results section. |
|  |  | *Case-control study—*Report numbers in each exposure category, or summary measures of exposure |  |  |
|  |  | *Cross-sectional study—*Report numbers of outcome events or summary measures |  |  |
| Main results | 16 | (*a*) Give unadjusted estimates and, if applicable, confounder-adjusted estimates and their precision (eg, 95% confidence interval). Make clear which confounders were adjusted for and why they were included |  | The main results reported the ORs and P-values from SMR and IVW analyses for each SD increase in mTOR expression associated with reduced BMD. All models had no additional confounder adjustment (summary-level data analysis), and confidence intervals were specified. |
|  |  | (*b*) Report category boundaries when continuous variables were categorized |  |  |
|  |  | (*c*) If relevant, consider translating estimates of relative risk into absolute risk for a meaningful time period |  |  |

Continued on next page

| Other analyses | 17 | Report other analyses done—eg analyses of subgroups and interactions, and sensitivity analyses |  | PheWAS results showed that rs4845985 was associated with basal metabolic rate (β = –16.769, FDR = 2.21 × 10⁻²⁵); results remained largely unchanged after MR-PRESSO correction. IVW-MR and MVMR analyses revealed age-dependent associations of insulin and fatty acid levels with BMD. No significant differences in blood mTOR expression were observed between high and low BMD groups. Molecular docking suggested that arachidonic acid had the highest binding affinity to mTOR (–5.9 kcal/mol). |
| --- | --- | --- | --- | --- |
| Discussion | | | | |
| Key results | 18 | Summarise key results with reference to study objectives |  | Our MR analysis indicated that genetically higher mTOR expression is associated with reduced bone density and shows potential colocalization signals, consistent with previous experimental studies, supporting a potential causal role of mTOR in bone metabolism. |
| Limitations | 19 | Discuss limitations of the study, taking into account sources of potential bias or imprecision. Discuss both direction and magnitude of any potential bias |  | It was noted that blood expression may not reflect target tissue expression; the study is limited to European populations; metabolic factors were not fully modeled; and findings are based on genetic effects rather than short-term pharmacological interventions, so direct extrapolation is not possible. |
| Interpretation | 20 | Give a cautious overall interpretation of results considering objectives, limitations, multiplicity of analyses, results from similar studies, and other relevant evidence |  | The study assessed the effects of lifelong differences in gene expression, which do not directly equate to the effects of short-term pharmacological mTOR inhibition. |
| Generalisability | 21 | Discuss the generalisability (external validity) of the study results |  | As the study primarily used data from individuals of European ancestry, the conclusions should be cautiously extrapolated to other populations. In addition, only the association between mTOR expression and BMD in adults was assessed, leaving effects in children or adolescents unclear. The equivalence of genetic effects to pharmacological interventions remains to be investigated. |
| Other information | |  | | |
| Funding | 22 | Give the source of funding and the role of the funders for the present study and, if applicable, for the original study on which the present article is based |  | The funding sources had no influence on the study design, data analysis, or publication. |
