# Supplementary material for: Mendelian Randomization Revealed Potential of mTOR Inhibitors for Treatment of Osteoporosis: Evidence From GWAS and Transcriptome Data
Source: Int J Endocrinol. 2026 May 23;2026:5562148. doi: 10.1155/ije/5562148 (PMC13197831; doi:10.1155/ije/5562148)
Supplement: Supplementary file 1 — Supporting Information Supporting table 1: Baseline information for cited studies. Supporting table 2: SMR analysis and HEIDI test for genetically proxied mTOR and BMD. Supporting table 3: Information about genetic instrumental variables for mTOR (Cis‐eQTLs). Supporting table 4: Bayesian colocalization results for mTOR instrumental variables. Supporting table 5: Bayesian colocalization results for mTOR with heel BMD. Supporting table 6: PheWAS results of the top SNP (rs4845985) of mTOR. Supporting table 7: SMR analysis and HEIDI test for mTOR and energy metabolism–related traits. Supporting table 8: Genetic instrumental variables for mTOR‐related metabolic traits and total body BMD. Supporting table 9: Genetic instrumental variables for mTOR‐related metabolic traits and total body BMD (age over 60). Supporting table 10: Genetic instrumental variables for mTOR‐related metabolic traits and total body BMD (age 45–60). Supporting table 11: Genetic instrumental variables for mTOR‐related metabolic traits and total body BMD (age 30–45). Supporting table 12: Genetic instrumental variables for mTOR‐related metabolic traits and total body BMD (age 15–30). Supporting table 13: Genetic instrumental variables for mTOR‐related metabolic traits and total body BMD (age 0–15). Supporting table 14: Molecular binding energies between mTOR and five fatty acids. Supporting figure 1: Expression boxplots of GSE56814 and GSE56815 before (a) and after (b) the removal of batch effects. PCA plots for high BMD and low BMD groups (c). PCA plot of GSE56814 and GSE56815 (d). Supporting list: STROBE‐MR checklist for the study. [file Supplementary figure 1.pdf › Supplementary figure 1.pdf]

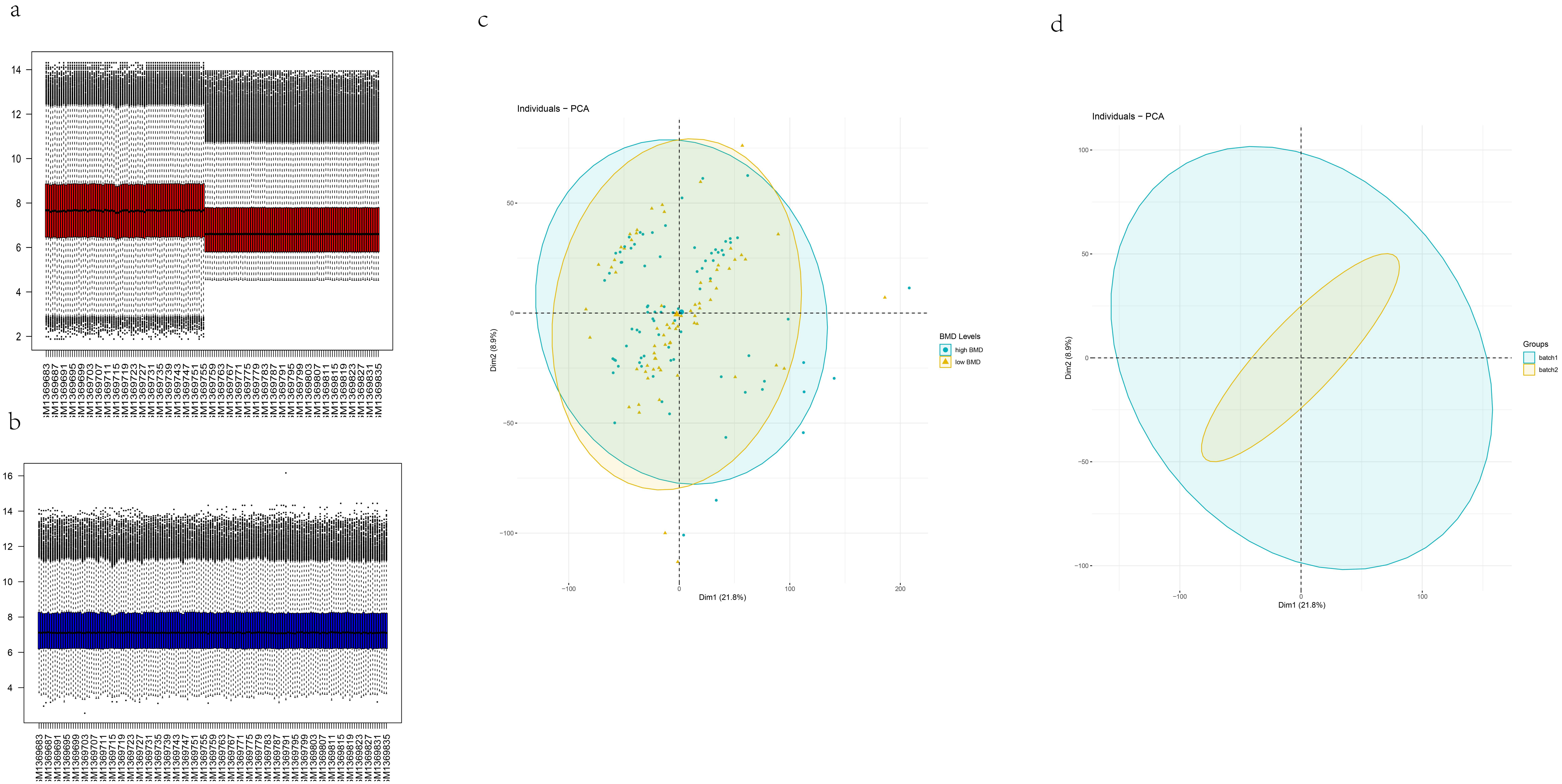

Expression boxplots of GSE56814 and GSE56815 before(a) and after(b) theremoval of batch effects.
PCA plots for high BMD and low BMD groups (c).
PCA plot of GSE56814 and GSE56815 (d).
